# Magnetite Nanoparticles Inhibit Tumor Growth and Upregulate the Expression of P53/P16 in Ehrlich Solid Carcinoma Bearing Mice

**DOI:** 10.1371/journal.pone.0111960

**Published:** 2014-11-06

**Authors:** Heba Bassiony, Salwa Sabet, Taher A. Salah El-Din, Mona M. Mohamed, Akmal A. El-Ghor

**Affiliations:** 1 Department of Zoology, Faculty of Science, Cairo University, Giza, Egypt; 2 Nanotechnology & Advanced Materials Central Lab, Agriculture Research Center, Giza, Egypt; Rutgers - New Jersey Medical School, United States of America

## Abstract

**Background:**

Magnetite nanoparticles (MNPs) have been widely used as contrast agents and have promising approaches in cancer treatment. In the present study we used Ehrlich solid carcinoma (ESC) bearing mice as a model to investigate MNPs antitumor activity, their effect on expression of p53 and p16 genes as an indicator for apoptotic induction in tumor tissues.

**Method:**

MNPs coated with ascorbic acid (size: 25.0±5.0 nm) were synthesized by co-precipitation method and characterized. Ehrlich mice model were treated with MNPs using 60 mg/Kg day by day for 14 injections; intratumorally (IT) or intraperitoneally (IP). Tumor size, pathological changes and iron content in tumor and normal muscle tissues were assessed. We also assessed changes in expression levels of p53 and p16 genes in addition to p53 protein level by immunohistochemistry.

**Results:**

Our results revealed that tumor growth was significantly reduced by IT and IP MNPs injection compared to untreated tumor. A significant increase in p53 and p16 mRNA expression was detected in Ehrlich solid tumors of IT and IP treated groups compared to untreated Ehrlich solid tumor. This increase was accompanied with increase in p53 protein expression. It is worth mentioning that no significant difference in expression of p53 and p16 could be detected between IT ESC and control group.

**Conclusion:**

MNPs might be more effective in breast cancer treatment if injected intratumorally to be directed to the tumor tissues.

## Introduction

Cancer is a major health problem causing mortality despite the discovery of several novel anticancer drugs [1]. Recently; the use of nanomaterials with diameters less than 100 nanometers has led to significant advances in *in vitro* and *in vivo* diagnosis and treatment of many diseases as cancer [2]. Several studies revealed that NPs may show different effects according to size, shape, composition, surface area, coating and aggregation state [3]. Iron oxide nanoparticle is the only metal oxide nanoparticles approved for clinical use for diagnosis of cancer [4], [5]. Of all the iron oxide species; magnetite nanoparticles (MNPs or Fe_3_O_4_) has the most significant applications in biomedical research because of their low toxicity and biocompatibility to human tissue [6]–[8].

Coating of MNPs is essential to reduce their aggregation, improves their distribution and stability, protects their surface from oxidation, increases the blood circulation time and cellular uptake, reduces toxicity and provides surface for conjugation of drug [9]. MNPs loaded with daunorubicin are shown to be highly biocompatible and safe nanoparticles and may be suitable for the treatment of hematologic malignancies [10]. Also, uncoated MNPs and Dextran-MNPs have induced cell death and reduced proliferation of fibroblasts in vitro [11]. In addition, a clinical trial of MNPs on patients with prostate cancer revealed a decrease in prostate-specific antigen that is correlated with prostate cancer [12].

It was reported that nanoparticles can target molecules that control cancer progression including p53 [13]. p53 is a tumor suppressor gene located on chromosome 11 in the mouse and on chromosome 17 (17p13.1) in human [14]. p53 controls cell proliferation and apoptosis and has an important role in cancer treatment [15]. p16 is also a tumor suppressor gene and is located on chromosome 4 in mouse and on chromosome 9 (9p21.3) in human. p16 contains an alternate open reading frame (ARF) that specifies a protein functions as a stabilizer of p53, as it can interact with and sequester Mouse double minute 2 homolog (MDM2) protein that cause degradation of p53 [16]. The expression level of p53 and p16 is down-regulated in different types of cancer [17], [18]. In addition, p16 and p53 deficiency cooperate in tumorgenesis [18]. Only a single study reported that exposure to MNPs induced a dose-dependent cytotoxicity and increased p53 gene expression at mRNA level in cultured pheochromocytoma (PC12) cells [19].

In the present study, we assessed the distribution of MNPs coated with L-ascorbic acid in Ehrlich solid carcinoma (ESC) bearing mice injected intratumorally (IT) or intraperitoneally (IP). In addition, we evaluated the antitumor effect of MNPs with both types of injection. Moreover, our study aimed to give insight into the possible mechanism of application of nanoparticles in cancer treatment by investigating the expression of tumor suppressor genes, p53 and p16, in ESC bearing mice.

## Materials and Methods

### Preparation and characterization of MNPs

MNPs with 25.0*±*5.0 nm size; was synthesized by co-precipitation method using ascorbic acid reduction of FeCl_3_. 0.25 g of FeCl_3_ powder was dissolved in 25 mL sterile saline. Then, 0.6 g Na_2_CO_3_ powder dissolved in 10 ml sterile saline was added to FeCl_3_ solution drop by drop with continued stirring for 10 minutes; the solution turned viscous with brown color. Following the addition of 0.12 g powder of ascorbic acid with vigorous stirring for 15 minutes, the color of solution turned black, and magnetite nanoparticles capped with L-ascorbic acid were formed. Finally, we completed the solution to 50 ml with sterile saline [7], [20]. The solution was sterilized for 3 hours by UV to kill any bacteria. Physico-chemical properties of magnetite nanoparticles were characterized using High-Resolution Transmission Electron Microscope (HR-TEM, FEI, Tecnia G20), X-ray Diffraction (XRD, PanAnalytical, X'pert Pro), Vibrating Sample Magnetometer (VSM, Lakeshore 7410) and Particle size analyzer (Zeta sizer anano series'zs', Malvern, UK).

### Animal model

Study protocol was approved by the Institutional Animal Care and Use Committee (IACUC), Faculty of Science, Cairo University, Egypt (permit number: CUFS/F/Cell Biol./02/13). All the experimental procedures were carried out in accordance with international guidelines for care and use of laboratory animals.

Six week old Swiss female albino mice with body weight 25–30 g were obtained from animal house of National Cancer Institute, Cairo University, Egypt. Upon arrival, the mice were randomly transferred to plastic cages containing sawdust bedding, and allowed to acclimatize for two weeks before the start of the experiment. They were housed under the standard conditions of room temperature (22–24°C), humidity (45–65%) and light (12 hrs light/12 hrs dark, cycles) and received food and tape water *ad libitum*.

### Tumor induction, treatment and sample collection

Murine Ehrlich Ascitis Carcinoma bearing mouse was obtained from National Cancer Institute, Cairo University (Giza, Egypt). Mice were randomly divided into six groups, six mice/group. Group1 was reference control. Groups 2 and 3 were injected with MNPs IP and IM respectively. Group 4 was injected with Ehrlich tumor only. Group 5 and 6 were injected with Ehrlich tumor and injected with MNPs IP and IT respectively. Groups that were not injected with MNPs were injected with saline. Mice of groups 4, 5 and 6 were implanted with 0.2 ml of Ehrlich tumor cell suspension (containing about 2×10^6^ viable cells) IM in the thigh of the left hind leg. Once solid tumor appeared on the day 14; mice were injected with 60 ppm of MNPs day by day. Tumor size was measured weekly using Vernier caliper. The following formula was used to estimate the tumor weight: Tumor weight (mg) * = * Length (mm) *×* (width (mm))^2^/2 [20]. After 14 injections; animals were anesthetized using sodium thiopental (0.5%) and were sacrificed by cervical dislocation. Tissues were collected, stored at −80°C for subsequent analysis.

### Measurement of iron content in muscles and ESC using ICP

Muscle and tumor samples weighing 52–950 mg (average 260 mg) were prepared for quantitative estimation of their iron concentration using inductively coupled plasma optical emission spectrometry ICP-OES (Thermo Scientific iCAP 7000) [21]. Tissues were dried in oven at 60°C for 12 hrs, and then the temperature was raised to 105°C for at least 6 hrs to determine the dry weight. Then the sample was placed in the muffle at 650°C for at least 12 hrs. Then the formed ash was digested with concentrated hydrochloric acid. The amount of iron was calculated from the linear portion of the generated standard curve.

### Histopathological examination of muscles and ESC

Autopsy samples from the solid tumor, thigh muscle were fixed in 10% formal saline for twenty four hours for pathological examination according the lab routine protocol. Briefly, tissues were dehydrated and embedded in paraffin wax. Sections of 5 µm thickness were obtained, dewaxed, rehydrated and then stained with hematoxylin and eosin (H&E) for microscopic examination [22].

### P53 and p16 genes expression analyses

#### RNA extraction and Semi-quantitative reverse transcription polymerase chain reaction (RT-PCR)

Total cellular RNA was extracted from frozen tissue samples of solid Ehrlich tumor and skeletal muscles using GeneJET RNA Purification Kit (Thermo scientific, USA) following the manufacturer's instructions and was stored at −80°c. RNA was treated with DNase I (Thermo scientific, USA) for removal of any remains of genomic DNA and then followed by EDTA treatment. First strand cDNA was generated from 1 µg of total RNA using RevertAid First Strand cDNA Synthesis Kit (Thermo scientific, USA).

Synthesized cDNA was used as template for amplification of p53, p16 genes by PCR [23]. Each gene was amplified in a separate 25 µL reaction using DreamTaq Green PCR Master Mix (2x) (Thermo scientific, USA). The reaction was performed using thermal cycler PCR (Techne TC-3000) for 35 cycles with annealing temperatures 59°C for p53 and GAPDH while 62°C was used for p16. The primers used for amplification are shown in Table 1. Glyceraldehyde 3-phosphate dehydrogenase (GADPH) was used as an internal control gene.

**Table 1:** Primer sequences for GAPDH, p53 and p16 mouse cDNAs.

Gene	Sense 5′–3′	Antisense 5′–3′	Product size (bp)
GAPDH	CAAGGTCATCCATGACAACTTTG	GTCCACCACCCTGTTGCTGTAG	496
p53	TGCTCACCCTGGCTAAAGTT	AATGTCTCCTGGCTCAGAGG	208
p16	TTGGCCCAAGAGCGGGGACA	GCGGGCTGAGGCCGGATTTA	200

#### Quantitative real time (qRT-PCR)

Synthesized cDNA was quantified using SYBR green-based real-time PCR and was detected with 7500 Fast system (Applied Biosystem 7500, Clinilab, Egypt). Total volume for each PCR reaction was 25 µL according to the manufacturer's protocol. The thermal cycling condition comprised an initial heat activation step at 95°C for 15 min followed by 35 cycles of denaturation at 95°C for 15 s, annealing and elongation at 55°C for 1 min. The primer sequences are the same used for RT-PCR (Table 1). Each sample was prepared as duplicate for each gene. The results of these genes were normalized to GAPDH. Dissociation curves were also conducted after amplification to ensure the reaction specificity. The amplification curve begins after the maximum baseline and the threshold was set in the exponential phase of the amplification curve. Results are reported as Mean ± Standard Error (SE) of relative change compared to the untreated control.

### P53 Immunohistochemical analysis

Immunohistochemical examination of p53 was performed using streptavidin-biotin method by Histostain-plus kit (Zymed, USA). Paraffin sections of ESC 5 µm-thick were dewaxed in xylene and rehydrated through graded alcohols. Then heat induced antigen retrieval in Tris –EDTA buffer was performed. Non-specific binding was blocked by 10% non-immune serum. Then tissues were incubated for 2 hrs in p53 antibody diluted 1∶50 with TBS. For negative control; tissues were incubated with TBS without primary antibody. The endogenous peroxidase activity of tissues was blocked with 3% hydrogen peroxide for 10 min (BioGenex, San Ramon, CA, USA). For visualization; tissues were incubated with 100 µl horse radish peroxidase (HRP) labeled rabbit or mouse secondary antibody and then DAB chromogen was added. For counterstaining, sections were stained with hematoxylin, then dehydrated and mounted. Sections were examined using light microscope (Olympus, CX41, Japan) to evaluate p53 immunostaining. Positive nuclei for p53 accumulation were stained brown. Expression of p53 was scored according to staining intensity and number of stained cells as follows: (score 0 or -ve) for no staining or very weak staining, (score 1 or +) for weak to moderate staining detected in 10–20% of carcinoma cells, (score 2 or ++) moderate to strong staining in 21–50% of cells and (score 3 or +++) for strong staining in >50% of cells [24], [25].

### Statistical analyses

The present data were analyzed by the aid of statistical package for the social sciences software (SPSS) version 18.0. Student's t-test or analysis of variance (ANOVA) was used to compare MNPs concentrations in tissues and genes expression among groups. P-value <0.05 was considered as statistical significance.

## Results

### Characterization of synthesized Magnetite nanoparticles

**Figure 1:**
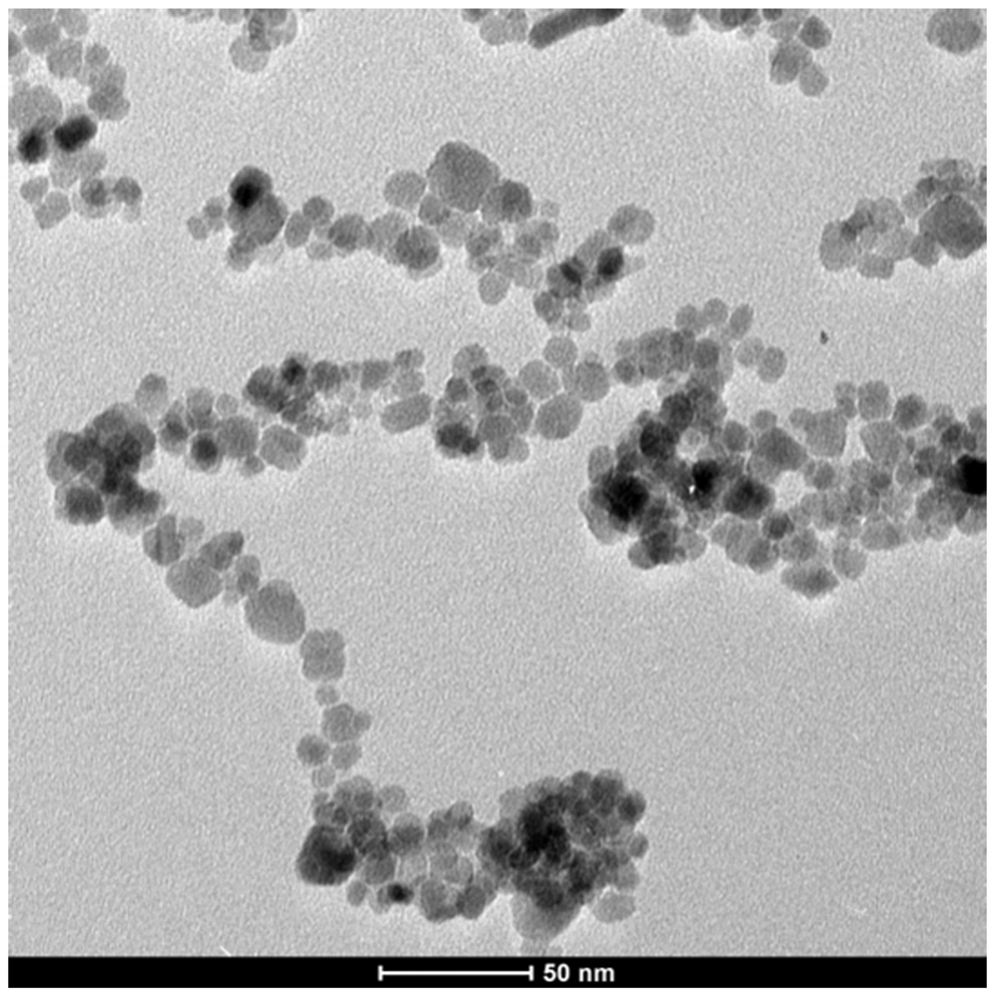
HR-TEM image of the prepared MNPs capped with ascorbic acid shows that particles have spherical shape with average size of 20.0±2.0 nm.

**Figure 2:**
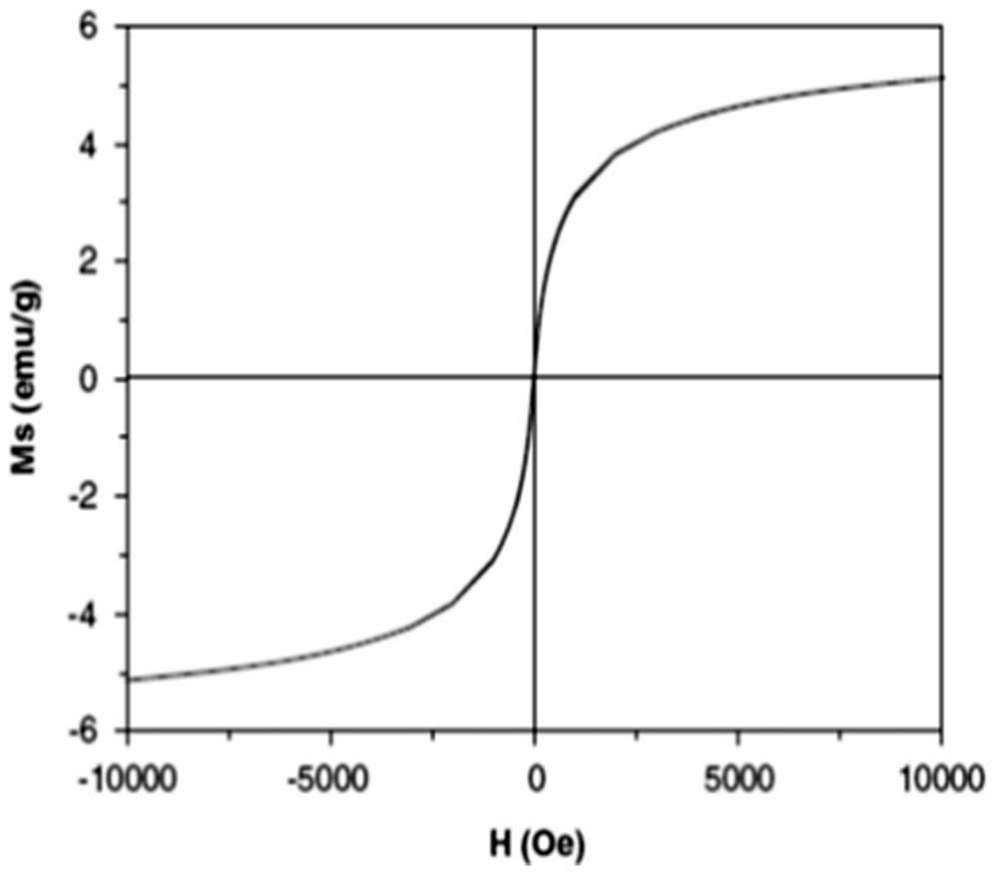
Hysteresis loop obtained from VSM measurements of synthesized MNPs capped with ascorbic acid at 300 K. It shows the magnetization (Ms) in an electromagnetic unit per gram (emu/g) in response to the magnetic field (H) in Oersted (Oe). MNPs saturation magnetization is 5.2 emu/g.

**Figure 3:**
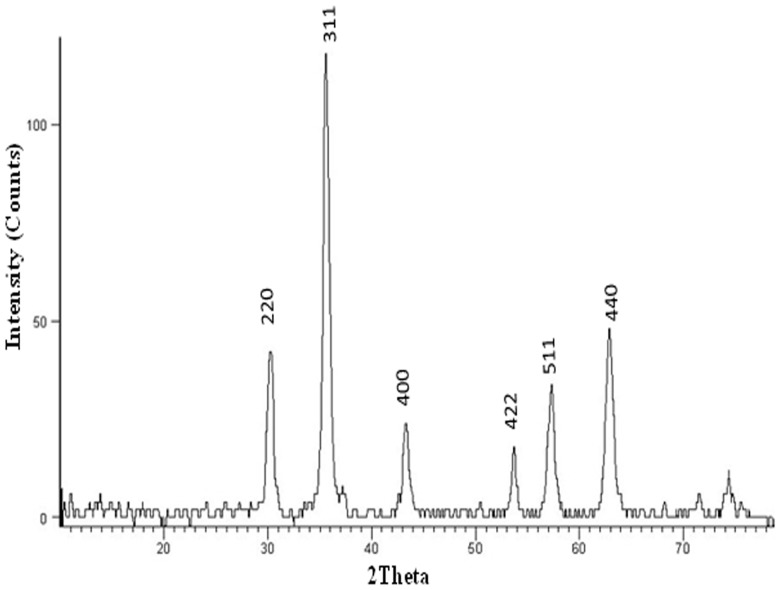
Graph represents the XRD pattern of synthesized MNPs shows the formation of Fe_3_O_4_ based on the comparison of their XRD patterns with the standard pattern of Fe_3_O_4_ (04-013-9808). The diffraction peaks are quite identical to characteristic peaks of the Fe_3_O_4_ crystal with the cubic spinal structure.

**Figure 4:**
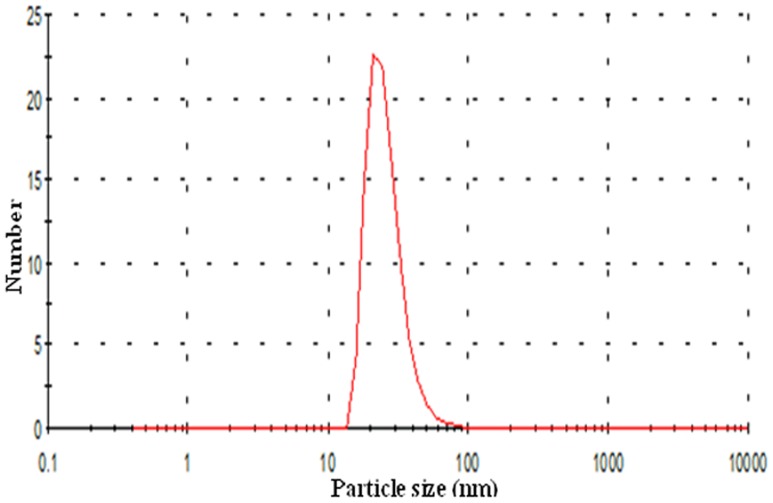
Graph showing particle size distribution by number for synthesized MNPs that was obtained by particle size analyzer. As shown from the observed peak the size of MNPs is 25.8 nm.

Magnetite nanoparticles capped with L-ascorbic acid (Vitamin C) were synthesized. The HR-TEM image of the synthesized magnetite nanoparticles shows that these particles has average size of 20.0±2.0 nm with spherical shape as shown in Figure 1. VSM generated a hysteresis loop from which the saturation magnetization (Ms) was calculated under magnetic field lower than 10,000 Oersted at room temperature as shown in Figure 2. The saturation magnetization of the product is 5.2 emu/g. The small saturation magnetization in our case is most likely attributed to the much smaller size of Fe_3_O_4_ nanoparticles and the existence of surfactants (C_6_H_6_O_6_) on the surface. XRD phase analysis confirms the phase formation of MNPs as shown in Figure 3. Particle size analyzer shows that size of synthesized MNPs is 25.8 nm (Fig. 4).

### Iron distribution in normal muscle and ESC tissues

**Figure 5:**
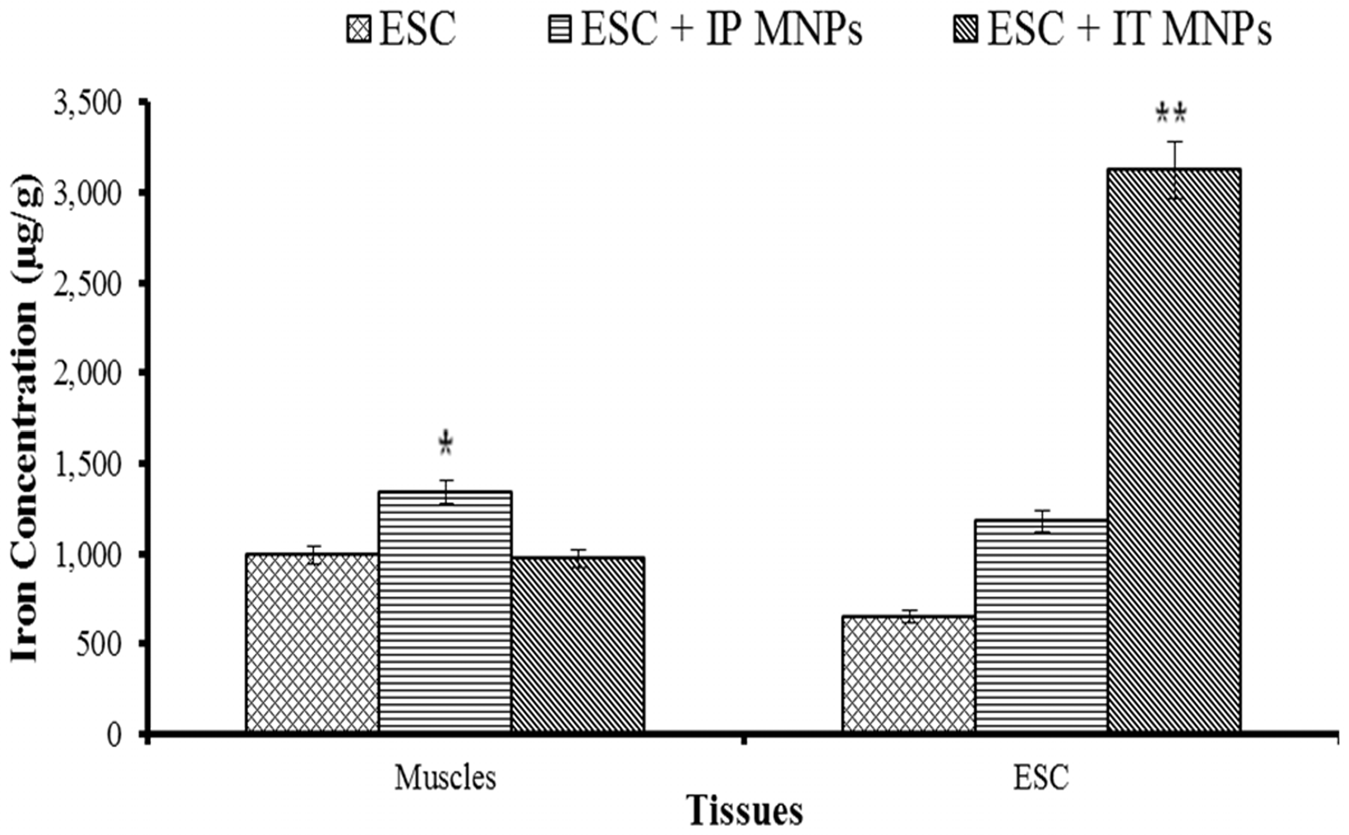
Bars represent distribution of iron in skeletal muscles and ESC tumor tissue of Ehrlich bearing mice groups. Asterisks indicate a statistically significant difference compared with other groups.

Magnetite nanoparticles bio-distribution was estimated in solid Ehrlich tumor and right thigh muscle of mice by ICP. Measurements per gram of dry weight of triplet samples are presented as Mean ±SE represented in Figure 5. The concentration of iron in the tissue was expressed as µg/g of dry weight.

For skeletal muscle tissues, IP injected mice have significantly highest iron concentration (1343.2±52.7) compared with other groups.

For tumor tissues; there is a significant difference in distribution of iron among groups. The highest concentration of iron (3120.5±68.7) in tumor tissues was recorded in IT injected group 6.

In addition, there is a significant difference in iron distribution between IT injected ESC and normal skeletal muscles.

### Anti-tumor effect of MNPs

Regarding the body weight of mice; no difference in any of the experimental groups was observed as compared to the control weights throughout the whole duration of treatment. That means that MNPs treatment had no effect on the body weights.

**Figure 6:**
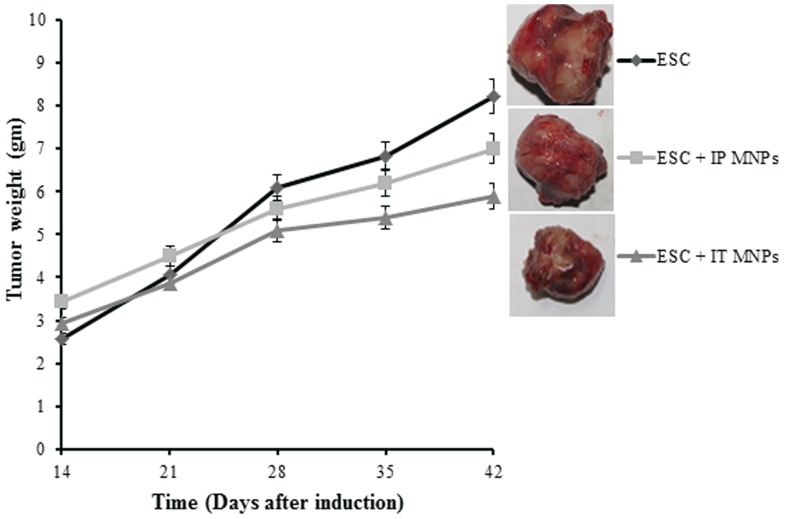
Antitumor effect of MNPs administration on Ehrlich solid tumor growth in mice. Ehrlich tumor bearing mice untreated with MNPs (♦), Ehrlich tumor bearing mice treated IP with MNPs (▪) and Ehrlich tumor bearing mice treated IT with MNPs (▴). Each point represents the mean±SE. n = 6. Representative images of tumor were shown for each group. Images were at the same magnification level.

To assess the anti-tumor effect of MNPs; MNPs were injected IP and IT to Ehrlich tumor bearing mice, and tumor growth rate was measured for up to 4 weeks (Fig. 6). From day 14 to day 21, the tumor size of both MNPs treated groups was similar to that of untreated group without significant difference. After that, an observed significant reduction in tumor growth of IT treated mice (group 6) and less reduction in tumor size was observed in IP treated mice (group 5), while untreated mice showed continuous tumor growth (group 4). The growth inhibition of solid Ehrlich tumor was found to be 47.35% and 37.1% for IT and IP treated animals, respectively.

### Histopathological effects of MNPs on normal skeletal muscles and ESC

**Figure 7:**
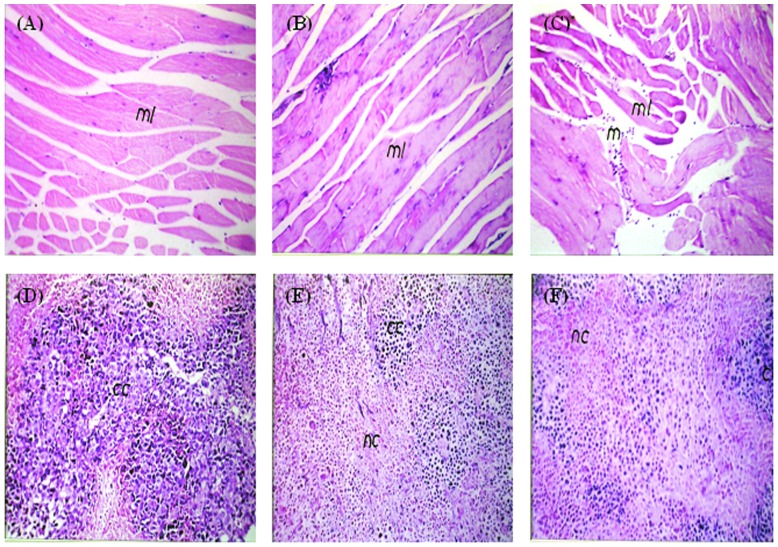
Photomicrograph of skeletal muscles and ESC of mice. (A, B) Muscles from control group and group injected IP with MNPs showing intact normal histological structure (ml). (C) Muscles injected IM with MNPs have focal inflammatory cells infiltration (m) in between the muscle bundles (ml). (D) Untreated tumor showing intact cancer cells (cc) occupying 90% of the skeletal muscle bundles with only 20% necrosis. (E) IP injected MNPs induced 40% necrosis (nc) of the injected Ehrlich tumor cells. (F) IT injected MNPs induced 60% necrosis (nc). H&E ×40

Microscopic examination of skeletal muscles stained with H&E showed no histopathological alteration in the muscle bundles treated IP with MNPs (Fig. 7B), similar to the normal control muscle tissues (Fig. 7A), While; focal inflammatory cells infiltration was detected in between the muscle bundles in muscles treated IM with MNPs (Fig. 7C).

Microscopic examination of ESC tissues stained with H& E showed that non-treated Ehrlich tumor occupied most of the skeletal muscle bundles as intact anaplastic area with few areas of necrosis in percentage of 20% per field (Fig. 7D). Necrosis increased (40%) in ESC treated IP with MNPs (Fig. 7E), while the most significant increase in necrosis (60%) was noticed in ESC treated IT with MNPs (Fig. 7F).

### MNPs increased levels of p53 and p16 genes expression in ESC tissue

**Figure 8:**
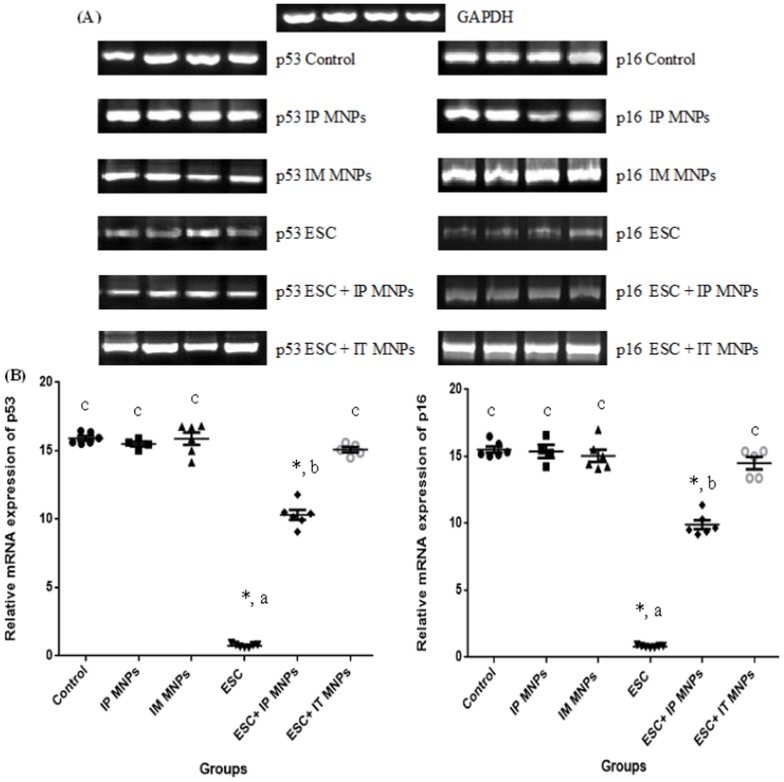
Comparison of effect of MNPs on the expression of p53 and p16 mRNA in ESC and skeletal muscles in our different six groups of mice. (A) Semiquantitative RT-PCR to amplify p53 (left), p16 (right) and GAPDH genes. Representative images are shown. (B) Real time PCR was performed to amplify p53 (left), p16 (right) and GAPDH genes. The mRNA ratios of p53 and p16 to GAPDH were calculated using the ΔΔCt method. Each bar represents mean ± SE of six independent experiment. Asterisks indicate statistically significant differences between this group and the control group (*p*<0.05). Both methods show that MNPs increases the expression level of p53 and p16 mRNA in Ehrlich tumor treated IP (group 5) and IT (group 6). Primers described in Table 1. - The same letter means that there is no significant difference between the two groups by using Duncan multiple comparison test (P>0.05). - The different letters means that there is a significant difference between the two groups by using Duncan multiple comparison test (P<0.05). - Asterisks indicate statistically significant compared with negative control using student t- test.

Changes of the gene expressions by MNPs were shown in Figure 8. We examined the expression of p53 and p16 genes at the mRNA level by conventional semiquantitative RT-PCR (Fig. 8A) and results were confirmed by real time PCR (Fig. 8B). Results showed that the expression levels of p53 and p16 were significantly (p<0.05) down-regulated in untreated ESC (group 4) compared with control muscles (group 1). Furthermore, the levels of p53 and p16 expression were significantly increased (p<0.05) in ESC treated IT with MNPs (group 6) when compared with ESC group and reached nearly the expression level in the control. In addition; significant increase in expression of p53 and p16 was noticed in ESC treated IP (group 5) when compared with ESC group, however, this increase still significantly less than the control level.

**Figure 9:**
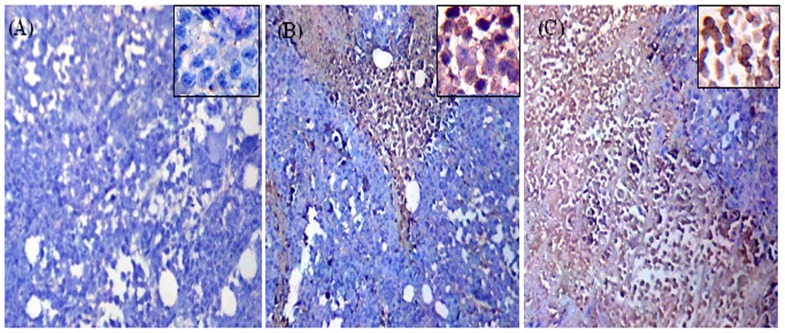
Photomicrographs represent immunohistochemistry staining of p53 expression of ESC sections from mice. **A**) Shows negative staining for p53 in ESC from untreated mice. **B**) Shows moderate expression of p53 (++) in ESC from mice received IP MNPs. **C**) shows overexpression of p53 (+++) in ESC from mice received IT MNPs. Magnification is 10X. Also higher magnified photo is shown for each group (40X).

Results of p53 gene expression were significantly confirmed by immunohistochemical analysis where ESC received IT MNPs highly expressed p53 (score 3, +++) (Fig. 9C) compared to moderate expression (score 2, ++) of p53 in ESC received IP MNPs (Fig. 9B) and negative staining for p53 in untreated ESC (Fig. 9A).

## Discussion

Magnetite nanoparticles have been widely used in biology and biomedicine, as they proved to be stable, non-toxic and noncarcinogenic [26]. Studies have highlighted the cellular responses induced by magnetite nanoparticles (MNPs) which include DNA damage, oxidative stress, mitochondrial membrane dysfunction and changes in gene expression [27]. However its effect depends on particle size, surface coating, exposure route and exposure duration [28].

Uncoated MNPs have very low solubility as it could be easily oxidized and aggregates together [29]. In previous studies, in order to improve their effectiveness and biocompatibility; MNPs were coated with amphiphilic coatings such as polyethylene glycol (PEG), polyethylene oxide, dextran, albumin, dendrimers or aspartic acid. They can also be bound to complex biological molecules such as antibodies, peptides, hormones or drug [30], [31].

In the current study we synthesized MNPs (25.0±5.0 nm) capped with L-ascorbic acid (vitamin C); a natural, safe and an inexpensive product; to enhance biocompatibility for in vivo application, increase cellular absorption of iron and decrease their toxicity. Our previous in-vivo toxicological studies indicated no mortalities in mice treated with MNPs using up to 60 ppm; the dose that we used in the present study and in a previous research for treating anemia [32].

After mice injection with MNPs, we compared iron distribution in ESC and skeletal muscle among groups of non-treated ESC, IP treated and IT treated groups. We found that MNPs accumulation in tumor tissues after IT was comparably higher than after IP administration. This proves that MNPs could successfully up taken by tumor tissues and more accumulated in tumor after IT injection. The observed slight increase in accumulated iron after IP treatment may be attributed to the reported ability of NPs to cross biological barriers such as blood vessel walls and cell membrane through endocytosis [33], [34]. The observed high accumulation of iron in tumor tissue after IT injection recommended the local administration which will be followed by tissue distribution as a result of the known enhanced permeability and retention (EPR) in tumor tissue and the perforated leaky tumoral blood vessels which allow molecules to accumulate passively in the tumor microenvironment [35].

Studying the antitumor effect of MNPs was one of our main objectives. All groups of mice injected with Ehrlich tumor cells (4, 5 and 6) were injected with approximately the same number of cells (about 2×106 cells) and showed the same tumor size at the beginning of experiment before treatment with MNPs without significant difference among groups. Interestingly, our results detected significant inhibition of tumor size, with a higher inhibition of ESC (47.5%) after IT administration of MNPs than after IP treated animals (37.1% inhibition). These results were confirmed by histopathological examination in which we found that tumor cells occupied most of the skeletal muscle bundles and only few areas of necrosis (20%) in non-treated tumor. While necrotic areas significantly increased in tumor groups injected with MNP, giving that the highest percentage of necrosis was observed in the IT treated group (60%) (Fig.7). A previous study showed that magnetic iron oxide nanoparticles caused hyper-thermia-mediated oncotic necrosis in head and neck cancer mouse xenograft model [36].

It's important to understand the possible molecular mechanism by which the MNPs induced tumor inhibition, as the novel cancer drugs rely on the molecular therapeutics which are designed against specific pathways [3], [4]. Thus, MNPs efficacy could be enhanced if coupled with a drug targeting the altered genes or proteins.

In this context, we examined the expression of the p53 and p16 genes that have an important role in inhibition of cell proliferation via cell cycle arrest and induction of apoptosis [37]. Our result illustrated that MNPs treatment IP or IT significantly increased the level of p53 and p16 mRNA in ESC in comparison to its level in the non-treated ESC (Fig.8). Also no change was observed in p53 and p16 expression in the IM or IP treated muscles. Similarly the protein level of p53 was significantly increased in ESC after IT injection of MNPs (Fig.9). Our results are consistent with an in vitro study reported that exposure to MNPs resulted in a dose-dependent cytotoxicity that was associated with increased p53 gene expression at mRNA level in cultured PC12 cells [19].

The possible explanation for the similar changes observed in p53 and p16 that both are tumor suppressor genes involved in many important physiological processes such as cell cycle arrest, gene transcription, DNA repair and apoptosis. They are frequently mutated and altered in most human cancers including breast cancer [15]–[17], [38], [39]. Normal breast epithelial cells induce p53-dependent apoptosis. p53 activation can be induced in response to stress. It eliminates and inhibits the proliferation of abnormal cells, so prevents neoplastic development [39].

p16 gene encodes protein that inhibits cyclin dependent kinases (CDKs) thus prevents phosphorylation of retinoblastoma protein (pRB) necessary for subsequent progression into the S phase of the cell cycle, so arrest cell cycle. p16 contains another alternate open reading frame (ARF) that specifies a protein inhibits mouse double minute 2 homolog (MDM2); a protein responsible for the degradation of p53 [16], [40]. So it stabilizes and preserves p53 activity in cell cycle arrest.

In conclusion our study has shown that MNPs was effective in tumor growth inhibition and enhanced the expression of p53 and p16 which direct cells to trigger programmed cell death by apoptosis in ESC cells. Moreover, IT injection of MNPs is preferable to direct these NPs to tumor tissues and indicates that MNPs may be useful in breast cancer treatment.
